# How Immunotherapy Has Changed the Continuum of Care in Hepatocellular Carcinoma

**DOI:** 10.3390/cancers13184719

**Published:** 2021-09-21

**Authors:** Giulia Martini, Davide Ciardiello, Fernando Paragliola, Valeria Nacca, Walter Santaniello, Fabrizio Urraro, Maria Stanzione, Marco Niosi, Marcello Dallio, Alessandro Federico, Francesco Selvaggi, Carminia Maria Della Corte, Stefania Napolitano, Fortunato Ciardiello, Erika Martinelli

**Affiliations:** 1 Oncologia Medica, Dipartimento di Medicina di Precisione, Università degli Studi della Campania “L. Vanvitelli”, 80100 Naples, Italy; giulia.martini@unicampania.it (G.M.); davide.ciardiello@unicampania.it (D.C.); fernando.paragliola@unicampania.it (F.P.); valeria.nacca@unicampania.it (V.N.); carminiamaria.dellacorte@unicampania.it (C.M.D.C.); stefania.napolitano@unicampania.it (S.N.); fortunato.ciardiello@unicampania.it (F.C.); 2 Chirurgia Epatobiliare e Trapianto di Fegato, A.O.R.N. Antonio Cardarelli, 80100 Naples, Italy; walter.santaniello@gmail.com; 3 Radiologia, Dipartimento di Medicina di Precisione, Università degli Studi della Campania “L. Vanvitelli”, 80100 Naples, Italy; fabrizio.urraro@policliniconapoli.it; 4 Malattie Infettive, Dipartimento di Salute Mentale e Fisica e Medicina Preventiva, Università degli Studi della Campania “L. Vanvitelli”, 80100 Naples, Italy; maria.stanzione@unicampania.it; 5 Epato-Gastroenterologia, Dipartimento di Medicina di Precisione, Università degli Studi della Campania “L. Vanvitelli”, 80100 Naples, Italy; marco.niosi@policliniconapoli.it (M.N.); marcello.dallio@unicampania.it (M.D.); alessandro.federico@unicampania.it (A.F.); 6 Dipartimento di Scienze Mediche e Chirurgiche Avanzate, Università degli Studi della Campania “L. Vanvitelli”, 80100 Naples, Italy; francesco.selvaggi@unicampania.it

**Keywords:** HCC, immune checkpoint inhibitors, multimodal treatment, biomarkers, AFP

## Abstract

**Simple Summary:**

HCC is a very aggressive disease and patients diagnosed in an advanced/metastatic setting obtain poor survival outcomes with standard treatments. In recent years, the introduction of immunotherapy strategies, such as immune checkpoint inhibitors as single agents and in combination with already approved local and systemic treatments, has strongly changed the therapeutic landscape of HCC. Soon, the discovery of novel potential immune targets, together with the understanding of potential biomarkers of resistance, will help to better define novel treatment opportunities for patients with HCC.

**Abstract:**

Hepatocellular carcinoma (HCC) is one of the leading causes of death worldwide. The use of local treatment, such as surgical resection, liver transplant, and local ablation, has improved the survival of patients with HCC detected at an early stage. Until recently, the treatment of patients with metastatic disease was limited to the use of the multikinase inhibitor (MKI) sorafenib with a marginal effect on survival outcome. New target approaches, such as the oral MKI lenvatinib in first-line treatment and regorafenib, ramucirumab, and cabozantinib in later lines of therapy, have demonstrated efficacy in patients with preserved liver function (Child–Pugh class A) and good performance status. On the other hand, the implementation of immune checkpoint inhibitors directed against PD-1 (nivolumab and pembrolizumab), PD-L1 (atezolizumab), and anti-CTLA4 (ipilimumab) in the management of advanced HCC has strongly changed the continuum of care of HCC. Future research should include the evaluation of molecular biomarkers that can help patient selection and provide new insight on potential combined approaches. In this review, we provide an overview of the clinical evidence of the use of immune checkpoint inhibitors in HCC, and discuss how immunotherapy has been implemented into the continuum of HCC care.

## 1. Introduction

Hepatocellular carcinoma (HCC) represents 90% of liver cancers and is one of the principal causes of death worldwide, with a steady rise of mortality rate [1]. Principal risk factors that might induce liver cirrhosis and are ultimately responsible for HCC disease are Hepatitis B or C viral chronic infections, alcohol abuse, obesity, diabetes, nonalcoholic steatohepatitis (NASH), nonalcoholic fatty liver disease (NAFLD), and certain inherited metabolic diseases [2]. HCC diverges from other solid tumors due to its origin in the context of cirrhosis. For this reason, a multidisciplinary approach with specialist figures, hepatologists, oncologists, surgeons, and radiologists, among others, is strongly required to guarantee correct patient management. HCC staging has been determined by different systems, with Barcelona Clinic Liver Cancer (BCLC) being the most common [3]. BCLC staging divides patients into stage 0/A with very early/early disease, stage B (intermediate) with multinodular HCC, stage C for advanced HCC amenable to systemic medical treatment, and stage D for patients with terminal disease only susceptible to palliative therapies [4]. Only patients included in stage 0/A are putative candidates for radical locoregional treatment modalities, such as surgical resection, radiofrequency ablation (RFA), and liver transplants, whereas transarterial chemoembolization (TACE) is reserved for stage B HCC [5]. Unfortunately, due to the lack of widespread screening programs that help detect the disease in early stages together with the stigma of risk factors (alcohol abuse, use of intravenous (IV) drugs) associated with the development of cirrhosis, HCC remains a silent killer. Since 2007, the mainstay of advanced HCC treatment has been limited to the use of a single drug, the oral multikinase inhibitor (MKI) sorafenib, with about three months increase in terms of survival and a series of related adverse events of importance [6]. Sorafenib is approved as a first-line strategy for patients with advanced disease, not amenable to locoregional treatments and transplant, with a Child–Pugh score of A or BCLC C criteria [7]. Recently, lenvatinib, another MKI, has received approval from the EMA and FDA and could be alternatively used in first-line settings, based on its non-inferiority activity compared to sorafenib [8]. Furthermore, in the field of targeted agents, regorafenib and cabozantinib failed to demonstrate non-inferiority to sorafenib in the first line but are approved for second-line treatment after sorafenib failure, as well as the anti-vascular endothelial growth factor receptor 2 (VEGFR2) antibody ramucirumab for patients with alpha-fetoprotein (AFP) ≥400 ng/mL [9,10,11].

In the last five years, the implementation of immunotherapy into the therapeutic armamentarium of HCC has strongly changed the medical approach to HCC patients. Therefore, immune checkpoint inhibitors (ICIs) have demonstrated promising results in Phase II and Phase III studies, and, to date, nivolumab and pembrolizumab have received accelerated approval by the Food and Drug administration (FDA) for second-line treatment of patients with advanced HCC after progression to sorafenib, while the combination of atezolizumab and bevacizumab has recently provided outstanding results in terms of overall survival (OS) and progression-free survival (PFS) in a first-line setting and has been recently approved by the FDA and European Medicines Agency (EMA) [12,13,14]. It is worth noting that several studies are currently investigating the combination of ICIs with different drugs, including targeted agents and chemotherapy, and novel immunotherapy strategies beyond ICIs are in current development. We herein provide an overview of the immune landscape of HCC, discuss the results of principal clinical trials that have led to currently approved immunotherapies and investigate potential immune biomarkers of response to improve outcomes in a disease that has always needed efficacious treatments.

## 2. Immune Landscape of Hepatocarcinoma Disease

The liver has a peculiar anatomy and fulfills functions including uptake of arterial and portal blood, filtration of gut pathogens, and excretion of toxic waste materials, a feature that leads to exposure to a high load of antigens [15]. This hepatic reticulo–endothelial system that comprises sinusoids, Kuppfer cells, and liver endothelial sinusoidal cells (LESC) activates innate T-cells through antigen presentation, causing a tolerogenic immune response in a physiological status [16]. It has been reported in several studies how the immune surveillance system could be damaged in the context of cirrhosis. This continuous inflamed status of the liver leads to the recruitment of cytokines and immune components, ultimate actors in neoplastic dysregulation in which HBV and HCV viruses could contribute to carcinogenesis development [17]. Immune tolerance that characterizes the evolution of HCC is regulated by innate and adaptive immune cells present in the immune tumor microenvironment (TME) such as CD4+ and CD8+ T-cells, dendritic cells (DCs), natural killer (NK) cells, myeloid-derived suppressor cells (MDSC), tumor-associated macrophages (TAMs) that express and up-regulate immune checkpoints on their surface as programmed cell death protein 1 (PD-1) and the cytotoxic lymphocyte protein 4 (CTLA-4) [18]. PD-1 is responsible for T-cell exhaustion and prevents T-cell activation by releasing cytotoxic mediators, and CTLA-4 impedes activation of T-cells by replacing CD28 in the interaction with CD80/86 ligands on antigen-presenting cells (APC) [19]. 

All these components of TME act with intricate processes as decreased tumor-associated antigen (TTA) recognition, accumulation of immune suppressive cells, and interaction between immune checkpoints and their ligands, leading to a final balanced immunotolerant status [20]. Moreover, the immunotolerance of liver cancer is accompanied by the release of several cytokines and regulatory factors, such as transforming growth factor (TGF)-β, which acts as an immunosuppressive factor [21]. 

## 3. Clinical Evidence of Immune Checkpoint Inhibitors in Hepatocarcinoma

Antigen presentation by major histocompatibility complex (MHC) is required for T-cell priming [22]. However, a cooperation between checkpoint molecules such as PD-1, PD-L1, CTLA-4, TIM-3, and LAG3 on T-cells and their ligands on antigen-presenting cells (APC) with the help of co-stimulatory molecules on receptors and ligands is needed for the activation of T-cells and finally establish a suppression of immune response against external antigens [23]. In a cirrhotic liver, the overexpression of suppressive checkpoint molecules with low production of cytokines in TME leads to a phenomenon called T-cell exhaustion, characterized by low immune activity and defective action against cancer [24]. Furthermore, the presence of a great number of antigens in liver TME complicates T-cell exhaustion. Hence, the use of ICIs has been explored in HCC to restore T-cell exhaustion and block HCC progression [25].

### 3.1. Nivolumab

The anti-PD-1 antibody nivolumab was investigated in the CheckMate 040 Phase I/II trial in 262 patients with advanced HCC, the majority (70%) after sorafenib failure. Response rate (RR) was 20% in the expansion cohort of the study and this result led to approval by the FDA for second-line treatment of HCC. The expansion phase of this study included a combination cohort in which patients received nivolumab alone or in combination with ipilimumab (anti-CTLA-4). In particular, patients were randomized 1:1:1 to receive nivolumab 1 mg/kg + ipilimumab at 3 mg/kg administered every 3 weeks, followed by nivolumab 240 mg every 2 weeks (Arm A), nivolumab 3 mg/kg + ipilimumab 1 mg/kg administered every 3 weeks, followed by nivolumab 240 mg every 2 weeks (Arm B) or nivolumab 3 mg/kg every 2 weeks + ipilimumab 1 mg/kg administered every 6 weeks (Arm C). Arm A (3 mg/kg every 3 weeks (4 doses), followed by nivolumab 240 mg every 2 weeks) reported the best results in terms of RR and overall survival rate at 12, 24, and 36 months of 61, 48, and 44%, respectively, and the combination obtained accelerated approval by the FDA [12].

By contrast, the phase III randomized CheckMate 459 trial (NCT02576509) which explored the efficacy and safety of nivolumab treatment in first-line treatment compared with sorafenib standard treatment did not show a statistically significant improvement in the primary endpoint of the overall survival (OS) (16.4 months versus 14.7 months in the nivolumab and sorafenib arm, respectively, Hazard Ratio (HR): 0.85; Confidence Interval (CI) 0.72–1.02; *p* = 0.0752), and in median progression-free survival (mPFS) (3.7 months vs. 3.8 months). However, RR was significantly higher in the experimental arm (15%) compared to 7% in the sorafenib control arm [26]. Although nivolumab treatment showed a clinical benefit in all the preplanned patient subgroups, with a favorable toxicity profile, the study did not change the standard of treatment for patients with naïve advanced HCC.

### 3.2. Pembrolizumab

Staying on the theme of anti-PD-1 ICIs, the KEYNOTE-224 Phase II trial reported data about the use of pembrolizumab after sorafenib failure. A RR of 17% and median OS of 12.9 months led to FDA accelerated approval of pembrolizumab in second-line settings [13]. However, results coming from the confirmatory randomized Phase III KEYNOTE240 trial failed to meet the prespecified co-primary endpoints of the trial OS and PFS with pembrolizumab compared with placebo in previously treated patients, although a survival improvement was obtained with respectively 16.9%, 13.9 months, and 3.0 months RR, OS, and PFS in the experimental arm, compared to 4.4%, 10.6 months, and 2.8 months in the control arm [27]. Even if not statistically significant, these data confirm the favorable risk-to-benefit ratio of the use of pembrolizumab after sorafenib failure, as compared with best supportive care.

### 3.3. Atezolizumab

The anti-Programmed Death-Ligand 1 (PD-L1) antibody atezolizumab was explored in a first-line setting in the Phase Ib GO30140 trial (NCT02715531) alone or in combination with the anti-VEGF monoclonal antibody bevacizumab. Arm A of the trial evaluated the combination of atezolizumab and bevacizumab every three weeks whereas Arm F randomized patients 1:1 to receive atezolizumab plus bevacizumab or atezolizumab as a single agent. The primary endpoint of Arm A, RR, was 36%, while PFS, the primary endpoint of Arm F, was 5.6 months with the combination and 3.4 months with atezolizumab single agent (HR: 0.55; 80% CI 0.4–0.74; *p* = 0.0108). Regarding safety profile, which was another co-primary endpoint of the study, a higher rate (68%) of treatment-related adverse events (TRAEs) was reported in atezolizumab plus bevacizumab combination versus 41% in the atezolizumab single-agent arm [28]. Subsequently, the Phase III Imbrave150 trial randomized 2:1 501 patients to receive a combination of atezolizumab plus bevacizumab or sorafenib as first-line treatment, respectively. The primary endpoints of the study were OS and PFS as per the independent review facility (IRF) assessed-response evaluation criteria in solid tumors (RECIST) 1.1.

Therefore, median OS was not reached in the atezolizumab plus bevacizumab combination that resulted in 13.2 months in the sorafenib group (HR: 0.58; 95% CI 0.42–0.79; *p* = 0.0006). OS rates were 84.8% and 67.2% with the experimental combination and the sorafenib arm obtained 72.2% and 54.6% at 6 and 12 months, respectively (HR: 0.58; 95% CI 0.42–0.79; *p* = 0.0006). Median PFS was 6.8 months in the combination arm and 4.3 in the sorafenib arm (HR: 0.59; 95% CI 0.47–0.76; *p* < 0.0001). The RR was 28% in the experimental arm versus 12% with control (95% CI 23–33 vs. 7–17, respectively, *p* < 0.0001), but reached 33% versus 13% (95% CI 28–39 vs. 95% CI 8–19; *p* < 0.0001) when evaluated per modified RECIST (mRECIST) criteria. Toxicities reported were more frequent with the experimental combination, with hypertension the most common grade 3–4 TRAE [14]. These data changed clinical practice with breakthrough approval of atezolizumab plus bevacizumab combination by the FDA and EMA as first-line treatment of patients with unresectable or metastatic HCC who had not received systemic therapy. In the same way, the updated OS analysis conducted with 12 months of additional follow-up from primary analysis confirmed the strong efficacy of atezolizumab + bevacizumab combination over sorafenib treatment, with median overall survival of 19.2 months for the combination versus 13.4 months for sorafenib. Moreover, updated results of RR were consistent with the ones from the primary analysis (29.8% per RECIST 1.1) with a higher rate of complete response (7.7%) than previously reported [29].

### 3.4. Anti-CTLA-4 Plus Anti-PD-1/PD-L1 Antibodies Combinations

The CTLA-4 immune receptor, expressed primarily on T regs and active T lymphocytes, binds CD80 and CD86 ligands, avoiding their interaction with CD28 thus limiting the antigen presentation process mediated by CD28, and dampening immune response [30]. The inhibition of CTLA-4 has been extensively studied in different solid tumors, such as melanoma and lung cancer [31,32]. Regarding HCC, the anti-CTLA-4 inhibitor tremelimumab has been explored as a single treatment or in combination with the anti-PD-L1 durvalumab as a second-line treatment strategy. In addition, a tremelimumab single agent has been investigated in patients with chronic HCV infection, in a Phase II clinical trial (NCT01008358), showing promising data with 17.6% RR, 76.4 disease control rate (DCR) and 6.48 months in terms of time to progression (TTP) [33]. After that, tremelimumab was investigated in combination with durvalumab in a randomized Phase II trial (NCT02519348) that enrolled 332 patients after sorafenib treatment failure, randomly assigned to the combination of tremelimumab at 300 mg + durvalumab 1500 mg, durvalumab single agent, tremelimumab single agent or tremelimumab at 75 mg + durvalumab. Safety, the primary endpoint of the study, was acceptable across all four arms, with the most common TRAE alanine aminotransferase (ALT) and aspartate aminotransferase (AST) increase, lipase increase, amylase increase, and diarrhea. Grade ≥3 TRAE in the 4 arms occurred in 37.8%, 20.8%, 43.5%, and 24.4%, respectively. RR by RECIST 1.1 and OS were secondary endpoints. Confirmed RRs (95% CI) were 24.0% (14.9 to 35.3), 10.6% (5.4 to 18.1), 7.2% (2.4 to 16.1), and 9.5% (4.2 to 17.9), respectively. OS was higher in tremelimumab 300 mg combined with the durvalumab arm with 18.7 (10.78 to 27.27) months, followed by tremelimumab single agent (15.11 (11.33 to 20.50) months), durvalumab single agent (13.57 (8.74 to 17.64) months), and tremelimumab 75 mg + durvalumab arm (11.30 (8.38 to 14.95) months) [34,35].

The efficacy and safety findings coming from the combination arm with tremelimumab at 300 mg have opened an avenue to the ongoing Phase III HIMALAYA trial (NCT03298451) of tremelimumab + durvalumab treatment compared to sorafenib in a first-line setting, where results are strongly awaited, as the two drugs were already granted orphan drug designation by the FDA in January 2020 [36]. 

## 4. Combining Immune Checkpoint Inhibitors with Targeted Agents

The development of HCC structure from dysplasia is a process influenced by proangiogenic factors such as angiopoietins, VEGF, transforming growth factors, basic fibroblast growth factors (bFGF), and platelet-derived growth factor (PDGF) that are secreted by TME and enhance tumor blood requirement from arteries to guarantee growth and metastatic spread. By contrast, tumor vessels are structurally imperfect and with a network of sinusoids that differ from hepatic sinusoids of a normal liver because they are more “capillarized” for the presence of a basement membrane and lack of fenestration [37]. Based on that, anti-VEGF strategies have represented a mainstay in HCC treatment for many years. 

The MKI sorafenib inhibits tumor angiogenesis and tumor-cell proliferation and was approved in 2007 for patients with advanced HCC, medical treatment naïve, with Child–Pugh A disease, after the results of the multicentric Phase III randomized SHARP clinical trial (NCT00105443) that enrolled 602 patients. Sorafenib treatment obtained an advantage in terms of OS compared to placebo group (10.7 months vs. 7.9 months; HR: 0.69; 95% CI: 0.55–0.87; *p* < 0.001) with survival rates at 1 year of 44% in the sorafenib group and 33% in the placebo arm [6]. Another chance for first-line treatment for advanced HCC is the MKI lenvatinib, which obtained FDA and EMA approval after the results of non-inferiority Phase III REFLECT trial (NCT01761266) for patients with absence of portal vein invasion, clear bile duct invasion, and 50% liver volume occupancy. Lenvatinib was not inferior to sorafenib treatment and had a tolerable safety profile [8].

Since then, treatment with other MKI molecules regorafenib and cabozantinib has been explored, without achieving statistically significant benefit in first-line treatment but in second-line treatment in the RESORCE (NCT01774344) and CELESTIAL (NCT01908426) Phase III trials, respectively. Interestingly, regorafenib treatment provided a median OS of 10.6 months, while the median OS of the sequence represented by sorafenib in first-line treatment and regorafenib in second-line treatment was 26.0 months, compared to 19.2 months with sorafenib and then placebo [9]. On the other hand, in the CELESTIAL trial, cabozantinib treatment in second or subsequent lines provided 10.2 months OS compared to 8 months with placebo (HR: 0.76; 95% CI 0.63–0.92; *p* = 0.005) and 5.2 months median PFS with cabozantinib and 1.9 months with placebo (HR: 0.44; 95% CI, 0.36–0.52; *p* < 0.001) [10].

**Figure 1:**
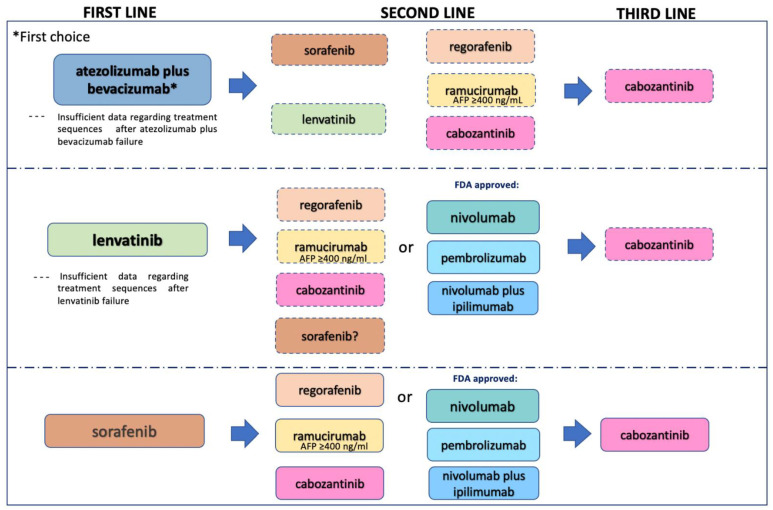
Different treatment scenarios in hepatocellular carcinoma.

Furthermore, the inhibition of angiogenesis in HCC was investigated with the anti-VEGF receptor 2 (VEGFR2) monoclonal antibody ramucirumab in the REACH trial (NCT01140347) and in the REACH-2 trial (NCT02435433). The REACH trial did not achieve statistically significant results but the analysis of a subgroup of “biomarker selected” patients with AFP ≥400 ng/mL showed a significant benefit in terms of OS (7.8 months in the ramucirumab arm compared to 4.2 months with placebo, HR: 0.67, 95% CI 0.51–0.90); based on that, the REACH-2 trial only included patients with AFP ≥400 ng/mL, obtaining significant OS advantage, confirming the previous trial analysis [11,38]. These data have opened the door to putative sequence strategies with MKI inhibitors in advanced HCC, as described in Figure 1.

Importantly, the tumor vessel-altered phenotype not only contributes to the new angiogenesis process that nourishes liver tumor but also determines a down-regulation of immune TME effectors such as CD8+ T-cells and a final immunosuppressive microenvironment in which T regs, MDSCs, and M2 polarized TAMs release immunosuppressive cytokines and finally block T-cells, NK cells, and DCs activation [39].

Based on this strong rationale, the combination of immune checkpoint inhibitors with anti-angiogenic drugs was examined in HCC and has proved effective strategies as per the case of the IMBRAVE150 trial of atezolizumab plus bevacizumab mentioned above. Furthermore, sintilimab plus anti-VEGF monoclonal antibody IBI305 safety and efficacy is currently being explored in first-line settings in the ongoing Phase II/III ORIENT-32 trial (NCT03794440). The same combination approach has been tried in second-line treatment with the anti-VEGFR-2 ramucirumab plus anti-PD-L1 durvalumab in the HCC cohort of the NCT02572687 Phase Ib trial. RR was 11% in the entire cohort but increased to 18% in the PD-L1 positive selected population, with a globally safe profile, suggesting future possible larger investigations in this setting [40].

Combining an anti-PD1/PD-L1 inhibitor to MKIs could represent another possible strategy under current development into different clinical trials (Table 1). Of note, lenvatinib in association with pembrolizumab as the first-line therapy determined in the KEYNOTE524 Phase Ib trial (NCT03006926) a 46 % RR per mRECIST (95% CI 36.0–56.3%) and 36% per 1.1 RECIST (95% CI 26.6–46.2%), with a median duration of response (DoR) of 8.6 months (95% CI 6.9 months to not estimable [NE]) per mRECIST and 12.6 months (95% CI 6.9 months-NE) per RECIST v1.1, median PFS 9.3 months per mRECIST and 8.6 months per RECIST v1.1 and median OS 22 months. Grade ≥ 3 TRAE occurred in 67% of patients; hypertension (36%), diarrhea (35%), and fatigue (30%) were the most common [41]. Similarly, the ongoing randomized Phase III LEAP-002 trial (NCT03713593) explores the efficacy of lenvatinib alone or in combination with pembrolizumab in patients with Child–Pugh A advanced HCC. Based on that, other promising data come from the Camrelizumab in Combination with Apatinib in Patients with Advanced Hepatocellular Carcinoma (RESCUE) nonrandomized, Open-label, Phase II trial. Seventy patients were treated with a combination of camrelizumab + apatinib in first-line and 120 in second-line settings. RR was 34.3, 95% CI (23.3 to 46.6) and 22.5%, (15.4 to 31), mPFS was 5.7 (95% CI, 5.4 to 7.4) and 5.5 months (95% CI, 3.7 to 5.6), and 12-month survival rate was 74.7% (95% CI, 62.5 to 83.5) and 68.2% (95% CI, 59.0 to 75.7), in first-line and second-line cohorts, respectively. Updated results with long-term follow-up recently presented by the authors showed remarkable data in terms of mOS 20.1 months for first-line treatment (95% CI, 14.9 to NE) and 21.8 months for second-line treatment (95% CI, 17.3 to 26.8), demonstrating promising efficacy with manageable toxicity profile (hypertension being the most common TRAE experienced by 74% of patients) [42,43]. Moreover, cabozantinib has been investigated in combination with nivolumab/nivolumab + ipilimumab in a cohort of the CheckMate 040 trial (NCT01658878) obtaining clinically meaningfully results in terms of RR (17% and 26%), DCR (81% and 83%), and mPFS (5.5 and 6.8 months) for doublet and triplet treatment, respectively. Although a high number of TRAE was observed in the triplet arm over the doublet arm, all the events were manageable. Furthermore, the COSMIC312 Phase III trial (NCT03755791) is currently investigating the efficacy of cabozantinib + atezolizumab compared with sorafenib in first-line settings. Final results are strongly awaited since preliminary data regarding the primary endpoint mPFS have shown a significant reduction in the risk of disease progression or death by 37% with the combination of cabozantinib + atezolizumab.

Other current studies are testing MKI association with immunotherapy as sorafenib in combination with nivolumab or pembrolizumab or regorafenib in combination with pembrolizumab in the NCT03347292 trial and avelumab in the REGOMUNE trial, respectively, and cabozantinib in combination with nivolumab/nivolumab plus ipilimumab in a cohort of the CheckMate 040 trial (NCT01658878) (Table 1). Another attractive strategy has investigated the use of chemotherapy in combination with ICIs for unresectable/metastatic HCC as the case of a Phase II trial testing camrelizumab plus FOLFOX-4)5-fluorouracil plus oxaliplatin) or GEMOX (gemcitabine plus oxaliplatin) chemotherapy regimens in advanced HCC or biliary tract cancer. In the HCC cohort RR was 26.5%, disease control rate (DCR) 79.4% and mPFS 5.5 months. Additionally, camrelizumab in combination with FOLFOX is currently being investigated in the Phase III NCT03605706 trial (Table 1).

## 5. Same Approach in a Different Setting

HCC has few chances of curability in advanced settings. Regarding the early stage defined by BCLC 0 or A criteria, different local approaches have provided an advantage in terms of efficacy and relapse-free survival, while chemotherapy or targeted agents have failed to demonstrate efficacy. In recent years, scientists have tried to harness the benefits of widely used locoregional approaches in combination with novel immune strategies. Giving the inflamed nature of liver tumor determined by its immunosuppressive phenotype, ICIs could prevent immune escape and avoid local recurrences after surgery. To this extent, anti-PD-1 and PD-L1 monoclonal antibodies are under investigation in adjuvant settings (Table 1). It is worth noting that the EMERALD-2 Phase III randomized trial (NCT03847428) is being conducted in patients with high risk of recurrence after surgery to study if durvalumab alone or in combination with bevacizumab provides better results compared to placebo in the adjuvant setting (Table 2).

## 6. Immunotherapy in Combination with Locoregional Treatment

As locoregional treatments represent the mainstay of HCC treatment, different strategies have explored their association with immunotherapy in early-stage disease settings. For example, TACE treatment reserved for patients with BCLC B HCC has been investigated in combination with sorafenib in the TACTIS trial, providing PFS of 25.2 months compared to 13.5 months with TACE alone *p* = 0.006) but unfortunately this advantage was not confirmed in terms of OS [44]. Then, immunotherapy has been associated with TACE treatment, based on the assumption of tumor necrosis induced by high intra-tumoral temperature that leads to the high release of TTAs and activation of liver-immune TME. Thus, several “ICI plus TACE” strategies are in progress and encouraging results from preliminary safety analyses justify further scientific development [45]. Moreover, novel studies are exploring if the TACE tissue damage could enhance the efficacy of anti-VEGF treatments as the EMERALD-1 trial (NCT03778957) of durvalumab and bevacizumab plus TACE, while the Phase III LEAP-012 is currently investigating pembrolizumab plus lenvatinib combination with TACE in incurable/non-metastatic HCC (NCT04246177).

Regarding other locoregional treatments, transarterial selective internal radiation therapy (SIRT) with Yttrium 90 microspheres has been investigated in BCLC B HCC as an alternative to TACE and current novel strategies are exploring its use in combination with ICIs, as pembrolizumab plus SIRT (NCT03033446), nivolumab plus SIRT (NCT02837029) in Asian patients and nivolumab after SIRT (NCT03380130). Furthermore, stereotactic body radiotherapy (SBRT) in association with pembrolizumab is being testing in the Phase II NCT03316872 study, after sorafenib failure. 

## 7. Strategies beyond ICI

Alternative immune strategies apart from ICIs in HCC are in current development. HCC disease is characterized by a high presence of TAAs, hence the use of vaccines that target TAAs being investigated. Principal targets of vaccine therapy in HCC have been AFP, glypican-3 (GPC-3), MAGE-1, human telomerase reverse transcriptase (hTERT), and NY-ESO-1. Interestingly, in a study that included 41 HCC and 24 non-HCC patients, the authors identified the capacity of circulating T-cells to recognize AFP-derived CD4+ T-cell epitopes and how anti-AFP CD4+ T-cell response was evident only in HCC subgroups with mild or elevated AFP serum levels, suggesting the potential use of anti-AFP cancer vaccines in HCC [46]. However, preliminary results coming from other analyses have provided mixed data, due to the small number of cases analyzed, deficient CD4+ helper T-cell support, or low numbers of antigens used in the vaccine. It is worth noting that encouraging results in terms of survival benefit have come from studies with glypican-3 (GPC-3)-directed vaccines [47]; clearly, further comprehensive analyses are warranted to strengthen these preliminary data. DCs have an important role in antigen presentation to T-cells in HCC, enhancing immune response; therefore, the use of DC vaccines has been tested in a Phase II trial showing promising benefit [48]. The use of viruses with high tropism to HCC cancer cells has been investigated in the HCC field as adenoviruses, vesicular stomatitis virus (VSV), vaccinia virus (VV), and Herpes Simplex Virus (HSV) [49,50]. To enhance anti-tumor efficacy, the virus genome is engineered with several genes that confer enhanced immunity [51,52]. Several preclinical data have provided encouraging results and some clinical trials with adenoviruses and vaccinia viruses modified with the deletion of TK and VGF and insertion of human GM-CSF have been conducted, showing some signs of activity, but the tolerability profile of these novel approaches needs to be confirmed in larger trials, including combination with approved ICIs [53].

Giving impressive results obtained with CAR-T-cell therapy in hematological malignancies, several preclinical trials have been conducted in solid tumors [54]. In HCC there are many obstacles to be removed such as the immunosuppressive microenvironment and the antigen heterogeneity that limits CAR-T-cell treatment efficacy, and preclinical and clinical studies are currently being conducted to further investigate this innovative strategy that represents a promising option for patients with HCC [55].

## 8. Correct Treatment Sequence after Implementation of ICI in the HCC Therapeutic Landscape

After the outstanding results of the IMbrave trial, atezolizumab plus bevacizumab combination has been considered to be the optimal strategy for first-line treatment. An important challenge is represented by the choice of the correct sequence for the treatment of later lines of therapy, as described in Figure 1. First, atezolizumab plus bevacizumab is the first treatment option for advanced disease, and sorafenib and lenvatinib MKIs, which have represented for years the only choice for first-line treatment, can now be considered for subsequent lines after atezolizumab bevacizumab failure. However, SHARP and REFLECT trials have investigated the use of sorafenib and lenvatinib in first-line treatment and, so far, only retrospective analyses of their use in second-line treatment have been published. Moreover, a limitation in the use of atezolizumab could be determined by the presence of uncontrolled autoimmune disease, while bevacizumab should be avoided in patients with high risk of bleeding. The other approved MKIs that are used in second-line treatment (regorafenib, ramucirumab, cabozantinib) have been investigated after sorafenib failure and there is a lack of evidence of their efficacy after atezolizumab plus bevacizumab failure. On the other hand, as the efficacy of the abovementioned MKIs has been largely demonstrated but there is no evidence of their direct comparison, international guidelines suggest their use even after atezolizumab plus bevacizumab progression. Another still-unanswered question is if an anti-PD-1 agent (nivolumab or pembrolizumab) could still be offered after progression of ICI-based therapy, as the anti-PD-L1 atezolizumab. In the same way, is it possible to treat patients with an anti-VEGF (ramucirumab) after bevacizumab failure? This strategy has proven effective in other diseases such as colorectal cancer but in HCC, data are very limited. Furthermore, so far only cabozantinib has been approved for third-line treatment, as 27% of patients enrolled in the CELESTIAL trial had already received two precedent treatment lines (sorafenib in the first line, ICI in the second). Nevertheless, there is no evidence of cabozantinib treatment after sorafenib and regorafenib or sorafenib and ramucirumab drugs. This and other questions remain unsolved. Future large clinical trials that include different treatment scenarios with combinations of different classes of agents in advanced settings could help define the correct treatment sequence of HCC.

## 9. Conclusions and Future Perspectives

**Figure 2:**
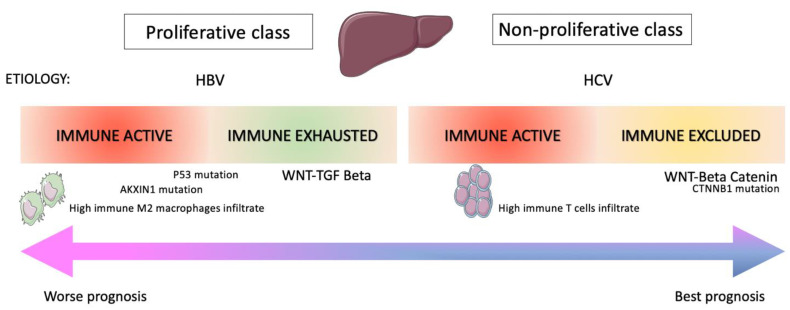
Molecular features of HCC immune classes.

HCC is a complex disease characterized by relevant immune-escape mechanisms that might be solved with immune strategies. However, the implementation of ICIs in the algorithm of HCC treatment has experienced a change in recent years as Phase III confirmatory clinical trials of pembrolizumab and nivolumab in second-line treatment unfortunately failed to reach the primary endpoint and updates regarding the accelerated approval by regulatory agencies are strongly awaited. Moreover, the use of ICIs in combination with different strategies such as target agents or locoregional treatments (TACE, SIRT) seems to provide better efficacy results, although safety matters such as hepatotoxicity still need to be addressed. Thus, identifying the correct sequence treatment and finding the “right place” for immunotherapy in the therapeutic armamentarium of patients with HCC represents a challenge. Furthermore, principal target agents and ICIs are still reserved for a subset of patients with HCC retaining a good performance status (Child–Pugh A, ECOG 0-1), a condition difficult to achieve most of all in second-line and subsequent lines of treatment and that sometimes could change during treatment. So far, we only have limited data of patients with Child–Pugh B with sorafenib and nivolumab. Therefore, analyses regarding real-world experience with ICI in HCC treatment could provide more comprehensive data of a heterogeneous population, to be taken into consideration to further extend the current indication of immune treatment in advanced HCC. Even though HCC is one of the principal causes of death worldwide, it is a complicated dynamic disease in which the immune system retains a strong role, and comprehensive knowledge is presently limited as immune resistance mechanisms are still unknown. In fact, the RR of patients that receive immunotherapy remains poor, and biomarker-guided strategies are strongly requested to increase the number of patients that could respond to treatment. To this extent, only ramucirumab represents so far a biomarker-guided treatment, while prognostic and predictive immune biomarkers are still unknown. PD-L1 expression is a well-recognized predictive biomarker for different solid tumors, but in HCC was assessed only in a retrospective analysis from the CheckMate 040 trial, in which no association was found with efficacy outcomes [12]. In this scenario, new data from preclinical trials, such as in vitro drug screening followed by validation in in vivo models, implementation into the immune therapeutic armamentarium of innovative tools such as liquid biopsy and genomic and transcriptomic signatures (Figure 2) that identify poor responders to immunotherapies could provide a “precision medicine”-based approach in the near future.

## Figures and Tables

**Table 1:** Principal ongoing clinical trials with immune checkpoint inhibitors in advanced HCC.

Study	Treatment (and Control)	Target	Setting/Target Patients	Start Date
NCT03638141	durvalumab and tremelimumab + DEB-TACE	Anti-PD-L1+ anti-CTLA-4	Intermediate stage/30 pts	2 October 2019
NCT04273100	PD-1 mAb + Lenvatinib + TACE	Anti-PD-1+ MKI	Intermediate stage/56 pts	14 November 2019
NCT04522544	Durvalumab and tremelimumab + SIRT or TACE	Anti-PD-L1+ anti-CTLA-4	Intermediate stage/84 pts	15 December 2020
NCT04268888	Nivolumab + TACE/TAE	Anti-PD-1	Intermediate stage/522 pts	8 May 2019
EMERALD-1 (NCT03778957)	Durvalumab +TACE versus durvalumab and bevacizumab + TACE versus placebo + TACE	Anti-PD-L1, anti-PD-L1 + anti-VEGF	Locoregional HCC/710	30 November 2018
LEAP012 (NCT04246177)	pembrolizumab + lenvatinib + TACE versus placebo + TACE	Anti-PD-1 + MKI	Intermediate stage /950 pts	22 May 2020
PETAL (NCT03397654)	Pembrolizumab following TACE	anti-PD-1	Intermediate stage/26 pts	28 January 2018
NCT03316872	Pembrolizumab + SBRT	Anti-PD-1	2nd line setting/30 pts	15 February 2018
NCT02837029	nivolumab+ SIRT	Anti-PD-1	Advanced HCC/27 pts	July 2016
NCT03033446 in Asian patients	nivolumab + SIRT	Anti-PD-1	Advanced HCC/40 pts	20 December 2016
NCT03380130	nivolumab after SIRT	Anti-PD-1	Advanced HCC/41 pts	11 September 2017
NCT02988440	Sorafenib + spartalizumab	MKI + anti-PD-1	Advanced HCC/20 pts	20 April 2017
NCT03347292	Regorafenib + pembrolizumab	MKI + anti-PD-1	1st line setting/57 pts	18 June 2018
Regomune (NCT03475953) Expansion cohort D	Regorafenib + avelumab in solid tumors	MKI + anti-PD-1	2nd line setting/482 patients	4 May 2018
Checkmate 040NCT01658878	Nivolumab; nivolumab + ipilimumab; Cabozantinib+ nivolumab; Cabozantinib + nivolumab + ipilimumab	Anti-PD-1, anti-PD-1+anti-CTLA-4; MKI + anti-PD-1; MKI + anti-PD-1 + anti-CTLA-4	Advanced HCC: uninfected HCC pts, HCV-infected HCC pts, and HBV-infected pts 659 patients	30 October 2012
NCT03764293	camrelizumab + apatinib versus sorafenib	MKI + anti-PD-1	1st line setting/510 pts	10 June 2019
COSMIC-312 (NCT03755791)	Cabozantinib atezolizumab versus sorafenib	MKI + anti-PD-L1	1st line setting/740 pts	10 December 2018
NCT03605706	Camrelizumab + folfox	Anti-PD-1	1st line setting/396 pts	31 May 2019
HIMALAYA (NCT03298451)	Tremelimumab + durvalumab versus sorafenib	Anti-CTLA4 Ab + anti-PD-L1	1st line setting/1504 pts	11 October 2017
LEAP-002 (NCT03713593)	Lenvatinib plus pembrolizumab versus lenvatinib	MKI + anti-PD-1 versus MKI	1st line setting/750 pts	31 December 2018
NCT03228667	N-803 + Durvalumab + PD-L1 t-haNK	Anti-PD-1/PD-L1	PD to ICIs/145 pts	11 December 2018

PD-1: programmed cell death-1, PD-L1: programmed cell death-ligand1, MKI: Multikinase inhibitor, VEGF: vascular endothelial growth factor; SBRT: Stereotactic Body Radiation Therapy, SIRT: Selective Internal Radiation Therapy, TACE: Transcatheter Arterial Chemoembolization, HCV: Hepatitis C virus, HBV: Hepatitis B virus, PTS: patients, CR: complete response, PD: progression disease, ICI: immune checkpoint inhibitor.

**Table 2:** Principal ongoing clinical trials with immune checkpoint inhibitors in adjuvant HCC.

Study	Treatment (and Control)	Target	Setting/Target Patients	Start Date
EMERALD-2 (NCT03847428)	Durvalumab versus durvalumab + bevacizumab versus placebo	Anti-PD-L1 + anti-VEGF	Adjuvant setting/888 pts	29 April 2019
Keynote 937 (NCT03867084)	Pembrolizumab Versus placebo	Anti-PD-1	Adjuvant setting/ CR after surgery or local ablation/950 pts	28 May 2019
Checkmate 9DX (NCT03383458)	Nivolumab versus placebo	Anti-PD-1	Adjuvant setting/ CR after surgery or local ablation/530 pts	18 April 2018
IMbrave050 (NCT04102098)	Atezolizumab + bevacizumab versus active surveillance	Anti-PD-L1 + anti-VEGF	Adjuvant setting/662 pts	31 December 2019
Jupiter04 (NCT03859128)	Toripalimab versus placebo	Anti-PD-1	Adjuvant setting/ CR after surgery/402 pts	1 March 2019
